# International Committee on Systematics of Prokaryotes: minutes of the open plenary meeting, Thursday, 19 June 2025, via Zoom

**DOI:** 10.1099/ijsem.0.006874

**Published:** 2025-08-07

**Authors:** Aharon Oren

**Affiliations:** 1 The Institute of Life Sciences, The Hebrew University of Jerusalem, The Edmond J. Safra Campus, 9190401 Jerusalem, Israel

**Keywords:** International Committee on Systematics of Prokaryotes (ICSP), minutes, plenary meeting

## Abstract

An online open plenary meeting of the International Committee on Systematics of Prokaryotes (ICSP) was held on 19 June 2025 via Zoom. To comply with Articles 4(d) and 5(d) (1) of the statutes of the ICSP, the minutes of this meeting are published here.

## Full text

An open plenary meeting of the International Committee on Systematics of Prokaryotes (ICSP) was held on 19 June 2025 online via Zoom. According to Article 4(d) of the statutes of the ICSP [1], minutes of the plenary meetings of the ICSP are reported by publication in the *International Journal of Systematic and Evolutionary Microbiology* (*IJSEM*). According to Article 5(d), it is the task of the Executive Secretary of the ICSP to submit the minutes of the plenary meetings for publication in the journal. To comply with the statutes, the minutes of this meeting are presented below.

## Minute 1. Welcome and call to order

ICSP Chair E.R.B. Moore called the meeting to order at 09.00 CEST.

## Minute 2. Record of attendance

The meeting was attended by Full members and members of the Executive Board E.R.B. Moore (Chair), C.M. Manaia (Vice Chair), A. Oren (Executive Secretary), R. Hahnke (Member at Large), M. Sakamoto (Member at Large) and M. Göker (Secretary, Judicial Commission) [Judicial Commission (JC)]; ICSP Full members M. Birbir, S.N. Dedysh, B. Duim, P.-E. Fournier, K. Jangid, M. Joossens, M. Kostovski, J.-S. Lee, L.A. Maldonado Manjarrez, I.C. Sutcliffe, P. Svec, S.N. Venter and S. Ventura (member, JC); ICSP Co-opted members H. Christensen (Vice-Chair, JC), T. Eisenberg, M. Figge, M. Hamada, J. Heo, H. Imachi, P. Kuhnert, M.-S. Li, A.J. Martín-Rodríguez, M. del C. Montero-Calasanz (member, JC) and A. Sidarenka; JC members D.R. Arahal (Chair, JC) and P. Young; and R. Montalvo-Rodríguez (member, Subcommittee on the Taxonomy of *Halobacteria*), J. Pieters and O. Prakash (chair, Subcommittee on the Taxonomy of Methanogenic Archaea). Apologies were received from S.L.W. On (Secretary for Subcommittees on Taxonomy, ICSP Co-opted member), P. Nielsen (Treasurer, ICSP), P. Hugenholtz (ICSP Full member), R.R. de la Haba (ICSP Co-opted member) and C. Sánchez-Porro (member, Subcommittee on the Taxonomy of *Halobacteria*).

## Minute 3. Overview of activities of ICSP since the last plenary meeting (an outline of a report was circulated to ICSP members before the meeting)

E.R.B. Moore explained that this is the fourth plenary meeting of the current ICSP term and that the time of this meeting was set to accommodate the ICSP members from the New Zealand–Australia–China–India time zones. The minutes of the earlier meetings can be found on the ICSP website at https://www.the-icsp.org/index.php/minutes-reports-2023-2026. The last plenary meeting was held at the CNR, Research Institute on Terrestrial Ecosystems, Sesto Fiorentino, Italy, in conjunction with the IUMS 2024 Congress on 22 October 2024 as a hybrid in-person and online open meeting. The minutes of that meeting have been published [2]. The ICSP was active in the organization and presentation of the congress and organized a symposium, hosted by the ICSP (represented by EB-ICSP Chair, E.R.B. Moore) and the University of Queensland (represented by Professor and Vice-President of IUMS, P.R. Young). Three invited lectures were presented: the van Niel International Prize lecture by the 2020 Prize recipient, T. Woyke, and lectures from ICSP members, A. Oren, S. Ventura and M. Göker.

The ICSP members list for the 2023–2026 has been updated on the ICSP website (https://www.the-icsp.org/index.php/icsp-members); there are currently a total of 58 members from 39 different countries (34 Europe, 13 Asia, 4 Pacific, 4 Americas, 2 Middle East and 1 Africa), 36 Full members, 19 Co-opted members and 3 Life members.

Since the last ICSP Plenary Meeting, the Belgian Society for Microbiology changed its representative on the ICSP – Welcome, M. Joossens, University of Ghent; the Association for General and Applied Microbiology (VAAM) in Germany has reactivated its membership in the International Union of Microbiological Societies (IUMS) and delegated its representative to the ICSP – Welcome, R. Hahnke, DSMZ, Braunschweig; and the German Society for Hygiene and Microbiology (DGHM) has changed its representative on the ICSP – Welcome M. Göker, DSMZ, Braunschweig. Welcome also to A.J. Martin Rodríguez (Stockholm and Las Palmas de Gran Canaria), who has been co-opted.

Since the last plenary meeting, seven voting issues have been completed or are ongoing, including three amendments of the International Code of Nomenclature of Prokaryotes (ICNP), two Requests for an Opinion, one amendment of the ICSP Statutes and one Co-option of a new ICSP member.

The Officers of the Executive Board of the ICSP are listed at https://www.the-icsp.org/index.php/executive-board-ics-p. The ICSP Executive Board meets monthly online to ensure matters of relevance to ICSP and *IJSEM* are addressed. Minutes are available online at https://www.the-icsp.org/index.php/minutes-reports-2023-2026.

## Minute 4. Overview of activities of the Judicial Commission since the last plenary meeting

D.R. Arahal, Chair of the Judicial Commission, summarized its recent activities. Opinions 131 and 132 have been issued [3,4], and two Requests for Opinion are pending, to become Opinions 133 and 134 [5,6].

The list of members is available at https://www.the-icsp.org/index.php/judicial-commission.

The Chair has attended a consultation for granting an exception to Rule 30(3)(b) of the ICNP [7] about the evidence of availability of type strains in at least two culture collections in different countries. The petition, which was approved unanimously, enables the valid publication of the names *Minisyncoccus archaeiphilus* gen. nov., sp. nov., *Minisyncoccales* ord. nov., *Minisyncoccia* class. nov. and *Minisyncoccota* phyl. nov. [8].

## Minute 5. Update from the Treasurer

E.R.B. Moore presented a report for 2025 year to date, prepared by the Treasurer, P. Nielsen, who apologized for his absence. Recent expenses were related to the van Niel International Prize awarded to T. Woyke at the IUMS meeting in Florence and travel expense reimbursement to 5 *IJSEM* editors. On 4 June, the balance of the Sterling account was £107,040.36. The balance of the US$ account, which is independent of the IUMS and Microbiology Society accounts and holds the historic Skerman bequest, was $25,620.56, unchanged since August 2024.

E.R.B. Moore commented that applications for funds to support ICSP-related activities are always welcome.

## Minute 6. Update from the Publications Committee and the *IJSEM*

A meeting of the *IJSEM* Editorial Board with participation of staff members of the Microbiology Society was held on 3 June 2025 to discuss the changes in the workflow for the handling of submissions that include *Candidatus* taxa, due to the implementation of the new Section 10 of the ICNP. The nomenclature guidelines on the *IJSEM* website have been updated accordingly.

The List Editors (A. Oren, M. Göker, M. Kostovski) adopted a new format for the preparation of the Candidatus Lists to implement pro-validation of *Candidatus* names in accordance with Section 10 of the ICNP.

Candidatus Lists no. 6 is in press [9]; Candidatus List no. 7 will be submitted in the coming weeks.

## Minute 7. Revision of the ICNP

Emendations of Recommendation 6(7), Rule 64 and Appendix 9 Section D of the ICNP to regulate the formation of prokaryote names from personal names took effect on 3 January 2025 [10]. Implementation of the new Section 10, Rules 66–73 for further integration of *Candidatus* names into the ICNP started on 9 January 2025 [11]. The proposed emendation to Rule 8 to clarify the meaning of the term ‘stem’ in the ICSP [12] was approved by the ICSP, and an announcement was submitted for publication in the *IJSEM*.

The Editorial Board of the ICNP published the proposed 2025 Revision of the ICNP on 29 January 2025 [13]. The proposal will remain open for discussion on the Slack channel until 29 July. The Editorial Board will then propose an updated version based on the comments raised, and the ICSP members will vote on the document. In case of problems accessing the Slack channel discussion, E.R.B. Moore, M. Göker and A. Oren can assist.

## Minute 8. Revision of the ICSP Statutes

A ballot to vote for the proposed amendments to the ICSP Statutes [14] is ongoing. The ballot form and content are found online at https://doi.org/10.5281/zenodo.15283452. Voting members may vote on all proposed amendments at once, or vote separately on blocks of amendments covering different topics: giving IUMS Member Societies the right to appoint more than one Full Member to the ICSP, depending on their size; giving Life Members the same voting rights as Co-opted Members; miscellaneous clarifications, adaptations to modern practice, filling of gaps and other improvements of the wording; separation of powers between the legislative and judiciary branch, reduction of bureaucracy and increase in fairness of procedures; formal regulation of the work of Ad Hoc Subcommittees in analogy to the work of Subcommittees on Taxonomy; clarification and streamlining of debates, revisions and voting procedures; and extending the time that committed members can serve on the Executive Board and broaden the range of members who can serve as Members-At-Large. The ballot will close on 25 July. H. Christensen and P. Nielsen serve as the tellers. H. Christensen reported that as of 18 June, 35 (64%) of the 55 members eligible to vote had returned the ballot form; about one-quarter submitted separate votes on the above-mentioned blocks of proposed amendments.

E.R.B. Moore had received a message from S.L.W. On, stating that, in his opinion, it is important that minutes of meetings of Subcommittees on Taxonomy be published in the *IJSEM*, also in the future. It was clarified that under the proposed revised statutes, publication of such minutes in the *IJSEM* remains possible.

## Minute 9. The ICSP website and other online resources

E.R.B. Moore reported that the ICSP website (http://www.the-icsp.org) is regularly updated and includes the most recent minutes of the monthly meetings of the Executive Board. He requested the officers of Subcommittees on Taxonomy, Ad Hoc Committees and Working Groups to send him the minutes of their meetings to be posted on the ICSP website.

M. Göker explained the use of the Zenodo platform to post meeting minutes and other relevant documents online. Many ICSP reports, the Frequently Asked Questions document, meeting minutes etc. are already on the ICSP Zenodo page. Uploaded documents have a DOI number and are permanently stored. To upload documents on the platform, creating an account on Zenodo is necessary and is easy. If necessary, M. Göker may assist.

M. Göker reported on the survey sent to all ICSP members and subcommittee chairs on the use of social media. Voting turnout was high. Most members who use social media are users of LinkedIn. Therefore, a LinkedIn account was opened (https://www.linkedin.com/company/international-committee-on-systematics-of-prokaryotes), and all are encouraged to use it. The ICSP Twitter/X account was not deactivated, but it is no longer updated.

## Minute 10. Activities of Subcommittees on Taxonomy, Ad Hoc Committees and Working Groups

The ICSP website lists 19 Subcommittees on Taxonomy that are currently active (https://www.the-icsp.org/taxonomic-subcommittees). The status of the Subcommittee on *Pseudonocardiaceae* is not clear after its chair, M. Goodfellow, passed away in March 2024.

Since the last plenary meeting, minutes of subcommittee meetings have been published: a closed meeting of the Subcommittee on the Taxonomy of Methanogenic Archaea [15], a closed meeting of the Subcommittee on the Taxonomy of Mollicutes [16], closed meetings of the Subcommittee on the Taxonomy of *Campylobacter* [17], a closed meeting of the Subcommittee on the Taxonomy of *Chlamydiae* [18], a closed meeting of the Subcommittee on the Taxonomy of Rhizobia and Agrobacteria [19], and a closed meeting of the Subcommittee on the Taxonomy of *Leptospiraceae* [20].

A proposal was received to establish a new Subcommittee on *Candidatus* taxa; no further information has been received. A Subcommittee on the Taxonomy of the *Neisseriaceae* has been proposed to be reactivated.

M. Göker reported on the activities of the Ad Hoc Committee on Mitigating Changes in Prokaryotic Nomenclature. The Ad Hoc Committee published the minutes of its monthly meetings (https://doi.org/10.5281/zenodo.10909264), a document with guidelines on prokaryotic nomenclature (https://doi.org/10.5281/zenodo.13871707), the workflow for compiling a list of recommended names for bacteria of medical importance (https://doi.org/10.5281/zenodo.15320328), and a list of recommended names for bacteria of medical importance (https://lpsn.dsmz.de/text/glossary#list-of-recommended-names-for-bacteria-of-medical-importance). A report of the work of the Ad Hoc Committee was submitted for publication in the *IJSEM* [21].

R. Hahnke prepared an outline of the activities of the Working Group on Education and Outreach, which supported a ‘Give me a Name’ contest for Spanish high school students, organized by M. del C. Montero-Calasanz. This contest was organized in the spring of 2025 for the third time; 71 students in 16 groups participated. A. Oren served as a judge and gave a presentation about nomenclature to the students. The ICSP Executive Board approved a £150 prize to the winning group. Further information and pictures of the prize awarding ceremony will soon be posted on the ICSP website. M. del C. Montero-Calasanz encouraged other countries to participate in similar contests in the future.

The ICSP is considering a request from M. del C. Montero-Calasanz to support an interesting approach to bacterial and archaeal nomenclature to a wider audience, by combining nomenclature with ‘geocaching’: a ‘hide and seek’ game with description of bacteria isolated from important buildings, monuments, parks etc.

The Working Group on Type Strain Accessibility is addressing problems resulting from challenges presented by the Nagoya Protocol on Access and Benefit Sharing (ABS). E.R.B. Moore reported on issues resulting from local regulations from Brazil, India, South Africa, Costa Rica and China.

Access to type strains from Brazil remains strictly regulated, and there is no mechanism for third-party access from foreign culture collections. A meeting was held with Brazilian authorities with the participation of M.E. Trujillo (Editor-in-Chief, *IJSEM*), several ICSP members and J. Overmann (DSMZ, Braunschweig) on 19 December 2024.

India has, in October 2024, issued more severe restrictions than before, as reported by K. Jangid. Earlier restrictions issued by the National Biodiversity Authority of India were based on the 2016 Notification for deposition of microorganisms for novel taxa description and the Biodiversity Act of 2002, revised in 2003. M. Figge asked whether the restrictions started with the Biological Diversity Act of 2003 or in 2016 with the implementation of the Nagoya protocol guidelines by the Indian Government. A. Oren asked whether names of species with type strains originating from India and effectively published before 2016 can be validated. The current situation is not clear. R. Hahnke will consult with the DSMZ lawyer, who has extensive experience with Nagoya ABS issues, to confirm the situation with the restrictions on Indian genetic resources. Depending on the advice of the DSMZ lawyer, the Working Group will try to meet soon about the Indian situation, preferably before the next meeting of the ICSP Executive Board, and K. Jangid (Full member, ICSP) will be invited to join. A. Oren asked whether type strains isolated outside India and held in Indian culture collections can be shipped to customers abroad without restrictions. K. Jangid replied that there are no problems in such cases.

E.R.B. Moore mentioned that a recent Ph.D. graduate from South Africa, J Pieters, made an unsuccessful attempt to overcome the local bureaucracy and restrictions on the deposition of type strains originating from South Africa in foreign culture collections under conditions that enable validation under the Rules of the ICNP. The working group and the Executive Board will continue working on the issues of the South African restrictions and will enlist the help of J Pieters and the representative of the South African Society for Microbiology to the ICSP, S.N. Venter.

The DSMZ in Braunschweig has signed an agreement with the biodiversity authorities of Costa Rica, and the CCUG in Gothenburg is working on establishing a similar agreement, so that these culture collections can provide type strains originating from Costa Rica to third parties for research purposes only without the need for additional permissions.

E.R.B. Moore reported that, in the past years, there were some technical problems that prevented researchers outside China from ordering type strains kept in Chinese culture collections. The problems were probably due to confusion among the Chinese culture collection curators about local regulations. He has been in contact with two collections, trying to solve the problems.

## Minute 11. Update on the IUMS 2026 Conference, Lisbon, Portugal, 4–6 November 2026

C.M. Manaia, who is a member of the national organizing committee of the IUMS 2026 Conference, to be held in Lisbon in November 2026, announced that the current Executive Board of ICSP is already involved in the organization of a session. The new Executive Board, to be elected in mid-2026, will further deal with this, starting in September 2026.

## Minute 12. The EU HORIZON-BIODIV call

S. Ventura and E.R.B. Moore encouraged ICSP members residing in the European Union and in countries associated with it to submit proposals for participating in the EU-HORIZON-BIODIV call (Strengthening Taxonomic Approaches for Biodiversity). Details can be found at https://cordis.europa.eu/programme/id/HORIZON_HORIZON-CL6-2025-01-BIODIV-03. The September deadline for submission of proposals is very close. K. Jangid commented that delegates of the EU HORIZON program recently visited India to discuss possible collaborations of Indian scientists with EU partners, but they did not mention the BIODIV call.

## Closing matters

Oren reminded the Full members and the Co-opted members that their membership in the ICSP will expire on 31 March 2026. Full members who wish to continue their membership must obtain letters of appointment from their microbiological societies. A. Oren will send reminders in September–October. Arrangements for Co-opted members to renew their membership will be announced after the revised Statutes of the ICSP take effect (see Minute 8). As several officers of the Executive Board must step down in 2026 or cannot be re-elected in the same function, based on the newly proposed version of the Statutes, ICSP members may consider becoming candidates for the Executive Board.

## Date/time of the next plenary meeting

The next plenary meeting, to be held online, was tentatively scheduled to take place in January or February 2026.

The meeting was adjourned at 10.45 CEST.

## Abbreviations

ABS: Access and Benefit Sharing
ICNP: International Code of Nomenclature of Prokaryotes
ICSP: International Committee on Systematics of Prokaryotes
*IJSEM*: *International Journal of Systematic and Evolutionary Microbiology*
IUMS: International Union of Microbiological Societies
JC: Judicial Commission
